# Brain training using cognitive apps can improve cognitive performance and processing speed in older adults

**DOI:** 10.1038/s41598-021-91867-z

**Published:** 2021-06-10

**Authors:** Bruno Bonnechère, Malgorzata Klass, Christelle Langley, Barbara Jacquelyn Sahakian

**Affiliations:** 1 REVAL Rehabilitation Research Center, Faculty of Rehabilitation Sciences, Hasselt University, Hasselt, Belgium; 2 Department of Psychiatry and Behavioural and Clinical Neurosciences, University of Cambridge, Cambridge, CB2 0SZ UK; 3 Laboratory of Applied Biology and Neurophysiology, ULB Neuroscience Institute (UNI), Université Libre de Bruxelles (ULB), Brussels, Belgium

**Keywords:** Geriatrics, Dementia

## Abstract

Managing age-related decrease of cognitive function is an important public health challenge, especially in the context of the global aging of the population. Over the last years several Cognitive Mobile Games (CMG) have been developed to train and challenge the brain. However, currently the level of evidence supporting the benefits of using CMG in real-life use is limited in older adults, especially at a late age. In this study we analyzed game scores and the processing speed obtained over the course of 100 sessions in 12,000 subjects aged 60 to over 80 years. Users who trained with the games improved regardless of age in terms of scores and processing speed throughout the 100 sessions, suggesting that old and very old adults can improve their cognitive performance using CMG in real-life use.

## Introduction

According to the World Health Organization (WHO), the world population aged over 60 years will have doubled in number by 2050, with an estimated total of 2 billion people^1^. Clinically, normal healthy aging is associated with some progressive decline in cognitive domains, such as processing speed and executive function. A significant decline in cognitive function, particularly memory, which is an early symptom of dementia, can lead to mild cognitive impairment (MCI). Currently it is estimated that 50 million are living with dementia worldwide and nearly 10 million new cases occur every year, representing a serious public health problem^2^. As such, the WHO has suggested that preventing cognitive decline and dementia is a global mental health priority. In addition to impacting the patient, dementia also has a significant impact on the family and society in general. The economic cost has been estimated at €232 billion for European countries in 2015 and is expected to double by 2040^3^. Age is the biggest risk factor for the development of dementia^4^, and aging is associated with a decline of cognitive function^5^. However, dementia is not considered a normal sequela of aging and prevention should be the key strategy to mitigate the identified risk factors^6^. Non-pharmacological interventions such as physical exercise and cognitive interventions^7,8^ may offer an alternative to pharmacological intervention in delaying dementia-related functional decline.

Over the last decade, the accessibility and use of smartphones and mobile internet has quickly expanded around the globe. In parallel to this rapid growth, the industry of mobile apps is exploding. Health-related apps make up an important part of this market, and numerous apps have been developed to ‘train’ cognition and challenge the brain, such as the ‘How Old Is Your Brain’ games developed by Dr Kawashima^9^ in 2006 which pioneered the arrival of this type of application. Since its release, many studies have been carried out to evaluate the efficacy of cognitive training using commercial or specially-developed applications. In cognitively healthy people aged 65 or older, there was some evidence from the included studies to suggest that 12 or more weeks of computerized cognitive training may improve cognition^10^, Similar results were found in recent meta-analysis summarizing the efficacy of commercially available cognitive training in the healthy elderly^11,12^. Finally, in people with MCI, the currently available evidence could not determine whether or not cognitive training would prevent clinical dementia or improve or maintain cognitive function^13^. The results of these studies suggest that the use of cognitive games could be effective in training cognition if used prior to the onset of dementia. These results were confirmed in a 10-year longitudinal study following 2802 healthy older adults to assess the efficacy of three cognitive training programs (training memory, reasoning, or speed of processing) relative to a control condition. Processing speed training resulted in reduced dementia risk (hazard ratio [HR] 0.71 (95% CI 0.50–0.99), *p* = 0.049) compared to control, but memory and reasoning training generated no significant risk reduction (HR 0.79 (0.57–1.11), *p* = 0.18 and HR 0.79 (0.56–1.10), *p* = 0.16, respectively)^14^.

Previous studies have suggested a beneficial effect of structured cognitive training using commercially available applications in healthy older adults^11,12^ however, those were not real-life use, but instead well-controlled studies with standardized training programs (i.e., number of sessions, duration, frequency). Currently, the literature in older adults supporting the benefits of Cognitive Mobile Games (CMG) when used in areal-life use context is still limited. Therefore, the main objective of our study is to add to the current knowledge by evaluating the efficacy of 100 sessions of CMG used in real-life (independently and without specific guidelines on training frequency) in older adults and how the effect may vary as a function of age. Based on the results of one previous study^15^, we hypothesized that the rate of improvement in CMG performance would be slower in the oldest subjects. Since training frequency was not imposed, the time to perform the 100 sessions may vary between subjects and type of CMG. Therefore, we investigated whether there was an impact of the time needed to perform all the sessions on the performance.

## Results

### Time needed to perform all sessions

Since no particular guidelines are given in the app regarding the frequency of the training sessions, we first analyzed the number of days needed to reach the 100 sessions for each CMG. Results were right-skewed so we presented the median, p25 and p75 (results according to the age groups are presented in Supplementary Table S1): 503 (230; 750) days for *Square Numbers*, 614 (407; 810) days for *Memory Sweep*, 410 (226; 646) days for *Word Pairs*, 200 (79; 424) days for *Babble Bots*, 411 (237; 610) days for *Must Sort*, 411 (233; 616) days for *Unique* and 472 (297; 657) days for *Rush Back*. Statistically significant differences were observed for the different CMG (p < 0.001) and within the CMG for the different age groups for *Memory Sweep* (p = 0.013), *Babble Bots* (p < 0.001) and *Unique* (p < 0.001).

We then performed linear regression to determine if the duration of the training has an impact on the progress (expressed in percentage of the progress obtained between the first and the last session, negative coefficient indicates that the progress are lower when the duration of the training increases). For *Square Numbers*: β =  − 0.03%, SE = 0.005, p < 0.001, for *Memory Sweep*: β = 1.12e^−5^%, SE = 4.08e^−3^, p = 0.95, for *Word Pairs*: β =  − 0.17%, SE = 0.05, p < 0.001, for *Babble Bots*: β = − 0.11%, SE = 0.05, p = 0.016, for *Must Sort*: β = 1.09%, SE = 0.71, p = 0.12, for *Unique*: β = − 0.28%, SE = 0.09, p < 0.001 and for *Rush Back*: β =  − 0.14%, SE = 0.03, p < 0.001. Complete results are presented in Supplementary Table S2 and in Supplementary Figs. S1–S7. Since we observed statistically significant differences between age group for the duration and that the duration may influence the progress, we adjusted the analysis of the changes of scores and processing speed by the total duration of the training for each participants and CMG.

### CMG scores

First, we analyzed the results of the first session of training to evaluate the influence of age on initial CMG scores. Results are presented in Table 1. We observed a statistically significant linear decrease in scores with increasing participant age in all CMG: *Square Numbers* (*p*_linear trend_ < 0.001, ε^2^ = 0.016), *Memory Sweep* (*p*_linear trend_ < 0.001, ω^2^ = 0.02), *Word Pairs* (*p*_linear trend_ < 0.001, ε^2^ = 0.005), *Babble Bots* (*p*_linear trend_ < 0.001, ε^2^ = 0.012), *Must Sort* (*p*_linear trend_ < 0.001, ε^2^ = 0.009), *Unique* (*p*_linear trend_ < 0.001, ε^2^ = 0.007) and *Rush Back* (*p*_linear trend_ < 0.001, ε^2^ = 0.003).

**Table 1:** Number of subjects in each age group (n) and mean (SD) or median [IQR] scores for the different CMG according to the age of the participants for the first session of training.

Age	Cognitive Mobile Games													
	Square Numbers		Memory Sweep		Word Pair		Babble Bots		Must Sort		Unique		Rush Back	
	n	Score	n	Score	n	Score	n	Score	n	Score	n	Score	n	Score
60–64	4863	16,735 [13,910]	3157	31,687 (6260)	3543	2460 [2310]	1006	4530 [7600]	3538	3392 [2019]	3558	3330 [3010]	3553	11,350 [10,550]
65–69	3591	16,600 [13,975]	2833	30,872 (6101)	3559	2460 [2095]	1015	4110 [8170]	3543	3205 [2160]	3569	3240 [3560]	3556	10,750 [11,200]
70–74	3312	14,485 [11,730]	1885	29,931 (5439)	3537	2460 [2360]	1012	3695 [6738]	3565	3005 [2280]	3048	2930 [3890]	3549	10,300 [11,050]
75–79	1034	14,442 [10,968]	726	29,246 (5408)	1345	1960 [2480]	1004	3020 [5772]	1421	2865 [2445]	1449	2910 [4740]	1330	9850 [10,600]
≥ 80	527	13,340 [12,205]	368	28,576 (5880)	723	1960 [2680]	1005	3530 [6010]	848	2758 [2742]	802	2610 [4150]	734	10,300 [10,738]

**Figure 1:**
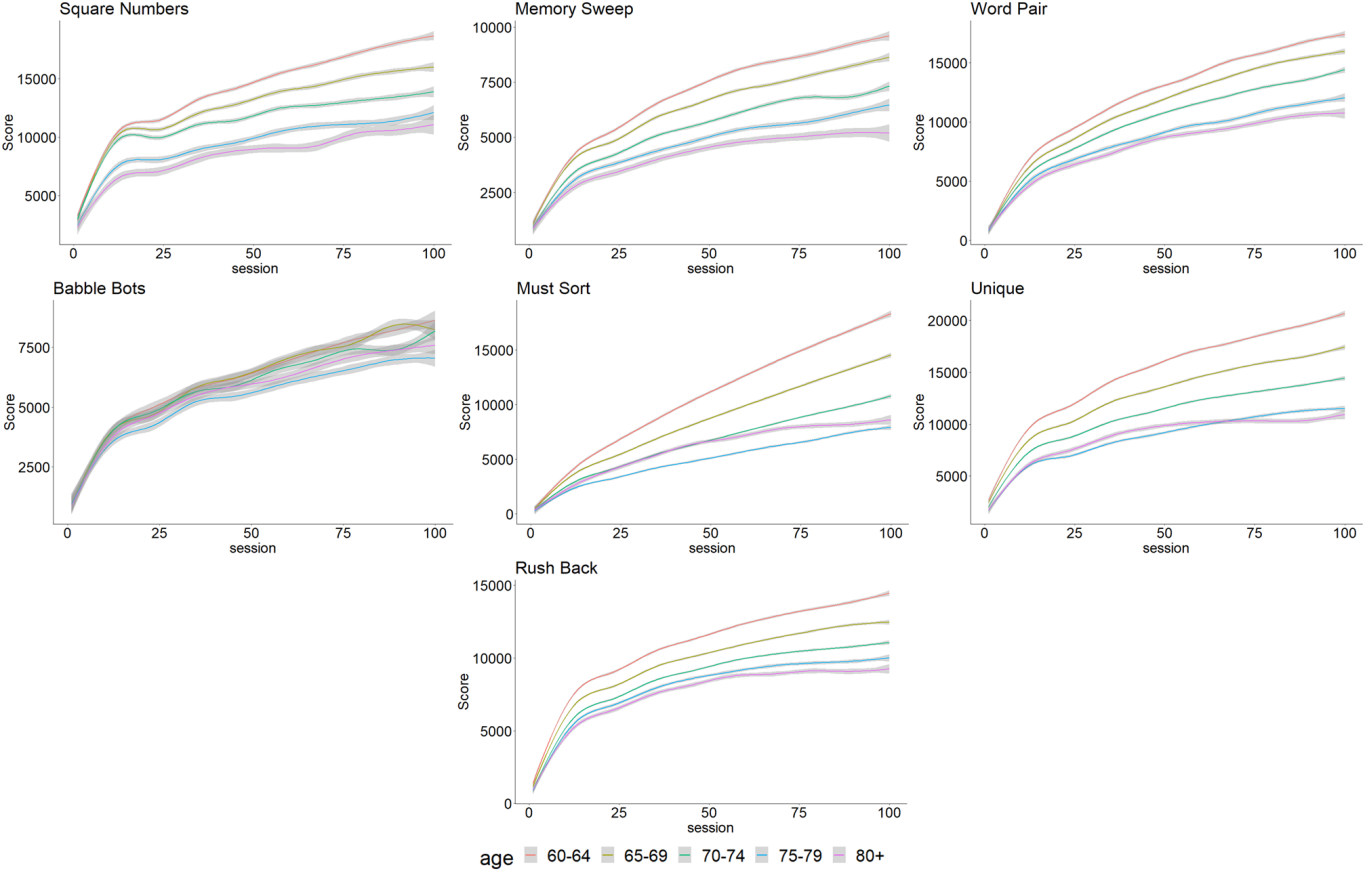
Time course of the scores for the 7 different CMG over the 100 sessions, grey bands are the 95% CI.

To analyze the time course of scores during the analysis, the results of the mixed models are presented in Table 2 and Fig. 1. We used likelihood ratio (LR) tests to determine if we needed to use models with or without interaction for each CMG and found significant results for all of them (*p* < 0.001). Therefore, an important outcome of this analysis is the interaction between training session and participant age group (results of the interactions in Table 2—complete results are available in Supplementary Table S3). As for the initial scores, we observed an interesting linear trend between age and session, indicating that all participants improved in all CMG but that the progress was slower in older participants (p < 0.001 for the 7 CMG).

**Table 2:** Results of the mixed models, β (SE) representing the change of score of the CMG per session training.

Age	Cognitive Mobile Games						
	Square Numbers	Memory Sweep	Word Pair	Babble Bots	Must Sort	Unique	Rush Back
60–64	108 (0.6)	76 (0.3)	143 (0.5)	61 (0.9)	172 (0.4)	148 (0.4)	101 (0.3)
65–69	89 (0.7)	63 (0.3)	130 (0.5)	61 (0.9)	131 (0.4)	123 (0.4)	87 (0.2)
70–74	75 (0.9)	54 (0.4)	114 (0.5)	53 (0.9)	96 (0.5)	100 (0.4)	78 (0.3)
75–79	63 (1.3)	45 (0.6)	93 (0.8)	48 (0.9)	69 (0.8)	80 (0.6)	73 (0.4)
≥ 80	61 (1.8)	38 (0.8)	88 (1.1)	50 (0.9)	87 (1)	81 (0.8)	68 (0.6)
*p*-value	< 0.001	< 0.001	< 0.001	< 0.001	< 0.001	< 0.001	< 0.001

Models are adjusted for the total duration of the training (Supplementary Table S1).

### Processing speed

As for the scores, first, we analyzed the results of the first session of training to evaluate the influence of age on initial CMG scores. Results are presented in Table 3. We observed a statistically significant linear increase in processing speed for the different CMG: *Square Numbers* (*p*_linear trend_ < 0.001, ε^2^ = 0.16), *Word Pairs* (*p*_linear trend_ < 0.001, ε^2^ = 0.09), *Must Sort* (*p*_linear trend_ < 0.001, ε^2^ = 0.32), *Unique* (*p*_linear trend_ < 0.001, ε^2^ = 0.17) and *Rush Back* (*p*_linear trend_ < 0.001, ε^2^ = 0.16).

**Table 3:** Median [IQR] processing speed (expressed as reaction time in ms) for the different CMG according to the age of the participants for the first session of training.

Age	Cognitive Mobile Games				
	Square Numbers	Word Pair	Must Sort	Unique	Rush Back
60–64	4371 [2391]	7478 [2059]	571 [240]	4121 [1472]	1175 [650]
65–69	4478 [2404]	7592 [2489]	609 [264]	4238 [1662]	1182 [685]
70–74	4450 [2158]	7775 [2852]	637 [283]	4396 [1685]	1226 [700]
75–79	5144 [2460]	8076 [2892]	665 [309]	4547 [2103]	1249 [695]
≥ 80	5811 [2505]	8086 [2880]	669 [380]	4643 [2459]	1215 [742]

**Figure 2:**
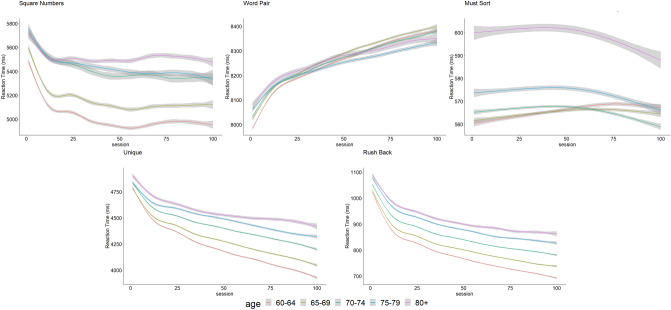
Time course of processing speed (measured as the reaction time) for the 5 different CMG over the 100 sessions, grey bands are the 95% CI.

When then evaluated the progress of the processing speed, the increase in game difficulty throughout the sessions must be taken into account. The time course of the processing speed over the 100 sessions, adjusted for difficulty levels and the total duration of the training, are presented in Fig. 2. The results of the mixed models are presented in Table 4. For *Word Pairs* we observed an decrease in the processing speed in all age groups. For *Square Numbers*, *Unique* and *Rush Back* there was a statistically significant increase for all participant age groups, however as for the score, the increase of the processing speed is more marked for younger participants. For *Must Sort*, only participants aged 70 and over presented a decrease in processing speed while the other age groups presented a slight increase, but in both cases the magnitude was relatively small (between + 0.09 ms [95% CI   0.07; 0.10]/session for the 60–64 age group and − 0.10 ms [95% CI − 0.14; − 0.06]/session for the 80+ group).

**Table 4:** Results of the mixed model, β coefficient [95% CI] representing the change of processing speed per session, results are expressed in ms.

Age	Cognitive Mobile Games				
	Square Numbers	Word Pair	Must Sort	Unique	Rush Back
60–64	− 3.12 [− 3.29; − 2.95]	3.60 [3.53; 3.65]	0.09 [0.07; 0.10]	− 7.0 [− 7.11; − 6.97]	− 2.51 [− 2.53; − 2.48
65–69	− 2.71 [− 2.90; − 2.52]	3.46 [3.41; 3.51]	0.04 [0.03; 0.05]	− 5.93 [− 6.01; − 5.87]	− 2.22 [− 2.24; − 2.20]
70–74	− 2.81 [− 3.20; − 2.41]	3.11 [3.06; 3.16]	− 0.05 [− 0.06; − 0.04]	− 4.98 [− 5.06; − 4.91]	− 2.07 [− 2.09; − 2.05]
75–79	− 2.59 [− 2.89; − 2.29]	2.38 [2.29; 2.47]	− 0.06 [− 0.08 ; − 0.04]	− 4.31 [− 4.42; − 4.20]	− 1.96 [− 1.99; − 1.92]
≥ 80	− 0.68 [− 0.89; − 0.48]	2.49 [2.36; 2.63]	− 0.10 [− 0.14 ; − 0.06]	− 3.68 [− 3.84; − 3.51]	− 1.77 [− 1.82; − 1.71]
*p*-value	< 0.001	< 0.001	< 0.001	< 0.001	< 0.001

Models are adjusted for the difficulty levels and the total duration of the training (Supplementary Table S1).

## Discussion

This study aimed to determine the efficacy of a cognitive training performed using CMG in real-life use on cognitive performance in older adults. First, we compared the baseline game scores per age group and observed that outcomes are sensitive to age-related cognitive changes, which is in line with the results of a previous study, where we showed that CMG scores are correlated with the cognitive abilities of older adults with and without cognitive impairments^16^.

When investigating the scores of the CMG, we observed statistically significant linear decreases with the increasing age of the participants, and conversely, a significant decrease in processing speed. These results are in accordance with neuropsychological and physiological data: aging is indeed related to a decrease in cognitive function^17^ and an increase in reaction time^18^. This observation supports that our outcomes are sensitive to age-related changes in cognitive function.

The literature also supports that basic numerical skills are preserved in healthy aging^19^ and that deficits may be associated with MCI^20^. The age-related differences in baseline scores we observed in *Square Numbers* are therefore probably not related to a decrease of numerical skills but may be explained by slowed reaction times and inhibiting abilities, both of which are known to be affected by aging^21^. Our study did not measure inhibitory processes directly, but *Must Sort* may be considered an indirect measure of inhibitory response. In *Must Sort*, we observed a linear decrease in scores as well as an decrease in processing speed with increasing participant age, both results are consistent with the aforementioned study^21^ and could explain why we observed age-dependent differences in baseline *Square Numbers* scores.

Though the changes in different cognitive abilities over the lifespan are relatively well-documented^22,23^, there is less evidence on the plasticity of these different cognitive functions across the lifespan^23–25^. Furthermore, it has not yet been established whether all cognitive functions can be trained or the extent to which progress can be achieved in healthy subjects of different ages^26^. These are both important questions in the field of cognitive training. Neuroplasticity is the ability of the brain to modify its structure and function for example under conditions of learning or compensation. We studied a healthy population and therefore the observed improvements are most likely due to training-induced plasticity rather than compensation. Previous studies have shown neuroimaging and neurotransmitter changes after cognitive training of working memory in healthy people^27–29^, that could ultimately lead to an increase of cognitive reserve^30^. However, it is possible given the age of the subjects that this may be a compensatory mechanism. For example the scaffolding theory of aging and cognition provides a theoretical model for the causes and the consequences of age-related compensatory neural activity^31^. According to this theory, scaffolding is conceptualized as the recruitment of additional circuitry that shores up declining brain function that has become inefficient. Despite the age-related alteration in different important brain structures (i.e., declining activity in the hippocampus, poor modulation of default network activity, amyloid deposition)^11,32^. Cognitive training or sustained engagement in challenging novel tasks like CMGs could enhance the development of scaffolding and as a result, confer protection and improvement in cognitive functions^33^.

We observed a clear linear trend for the analysis of the initial score, the same tendency was found for the time course of the scores, where all progress were smaller with increasing age. Those results confirm that even if the age-related cognitive decline is inevitable, lifelong trajectories of brain and cognitive functions are variable and stay plastic throughout the lifespan^34^.

For the next part of discussion, we will address the effect of training on each cognitive domain (see Table 5 for the different cognitive abilities trained by the CMG) in turn. Note that each CMG may train different cognitive abilities but for the sake of this discussion, we define the main component of cognition for each CMG.

**Table 5:** Instructions, cognitive abilities trained, scoring system of the CMG, and how processing speed is measured in each of the CMG included in this study.

CMG	Instruction	Main cognitive abilities trained	Time per CMG (s)	Total training time (min)	Scoring system	Processing speed
*Square Numbers*	Match the target shown on top by adding two or more number blocks	Quantitative reasoning Arithmetic Working memory	70	117	Base score for each correct answer with a speed related bonus (50 − (elapsed seconds × 5)) Streak up after 4 correct rounds, down after 5 incorrect	The processing speed is measured as the inverse of the average time to perform the different calculations
*Memory Sweep*	Memorize the positions of the highlighted tiles and remember their positions when gone	Attention Spatial memory Working memory	90	150	Points for each correct square (250) plus a base score for complete round Streak up (bonus) after 1 correct answer, down after 1 incorrect move	NA
*Word Pair*	Pair words according to a specific rule presented (similar, opposite)	Semantic access Vocabulary	90	150	Base score for correct round, streak up after 2 correct answers, down after 2 incorrect	The processing speed is measured as the inverse of the average time to pair the different words
*Babble Bots*	Create the maximal number of words of at least 3 letters with the 6 available letters	Word fluency Vocabulary	60	100	Points for letters in word multiplied by the word length, as the streak multiplier. Letter scores are localised to the region based on the Scrabble scoring system	NA
*Must Sort*	Sort the items correctly by tapping on the correct side	Response control Task shifting	45	75	Base score multiplied by streak multiplier, streak is incremented by correct answers and is reduced to 1 on incorrect or more than 5 s between answers	The processing speed is measured as the inverse of the average time between when the card appears and taping on the screen
*Unique*	Find the odd one out and tap on it	Visual attention Visual recognition	70	117	Baseline score per correct answer based on difficulty level. Delta is added to the baseline and becomes larger with consecutive correct answers (Baseline + (streak × delta)). Streak of 8 correct up, 6 down	The processing speed is measured as the inverse of the average time between when the objects appear and the discovery of the unique object
*Rush Back*	Memorize a shape, then decide if the next shape matches the one memorized	Sustained attention Visual recognition Working memory	45	75	One base score per difficulty level with a multiplier which goes up and down based on streak Streak up of 4 correct in a row but not changed during game play Bonus for end of game, current streak multiplier × bonus	The processing speed is measured as the inverse of the average time between when the card appears and the classification

### Arithmetic ability: Square Numbers

We observed a small but significant decrease in processing speed in Square Numbers over the course of the 100 sessions, even in the 80+ age group. The processing speed increases during the first 50 sessions then remain stable while the score of the games is continuously increasing, this seems to indicate that the speed is no longer decreasing but the participants are able to perform more complex tasks. There are, to the authors’ knowledge, no existing studies assessing the evolution of processing speed during arithmetic training in the older adults, with previous studies only investigating these outcomes in primary school students^35^ or young adults^36^. In both studies, the authors observed an improvement in subjects’ arithmetic abilities as well as processing speed. The results of the present study are consistent with these results and extend them to older adults.

### Word processing: Word Pairs

We observed an increase in *Word Pairs* scores throughout the sessions in every age group. This increase was greater for the younger participants. Word processing and literacy engagement along adulthood enable to maintain an efficient lexical processing^37^, which is reflected by the evolution of the scores observed in the current study indicating that semantic learning abilities are preserved even at advanced ages. However, concerning the processing speed, even after adjusting for the difficulty level, we observed an increase in all age groups during the training. *Word Pairs* and *Babble Bot* are the only two CMG using retrieval from long-term memory. Participants tended to recall common, more easily accessible items before unique, less accessible items, and this pattern was more prominent in older adults^38^. The words to pair become more difficult and less common as the training progresses, which may explain why, despite the adjustment, the time needed to associate these words increases significantly in the different age groups.

### Response control and task-shifting: Must Sort

It has been demonstrated that older adults experience more difficulties in task switching, coupled with infrequent and unexpected transitions from one task set to another^39^. Despite the highest costs to task shifting performance^40^, we observed that older participants were able to train this function, as exhibited by their significant improvements in processing speed. One potential mechanism that could explain this is a shift in cognitive control. Previous neuroimaging studies have indeed shown that older adults may switch from a proactive (e.g., anticipation) to reactive cognitive control strategy (e.g., *late-correction* mechanism) as a means of retaining relatively preserved behavioral performance in the face of age-related neurocognitive changes^41^. In the *Must Sort*, reactive control strategy is the most used mechanism.

### Visual attention: Unique

With regard to visual attention, it is widely accepted that aging is associated with the deterioration of vision and field of view^42^, and with a decrease in selective attention^43^. We observed that the time needed to find the unique object decreased in all age groups over time, which may indicate that this CMG is able to improve selective attention in older adults, or at least improve response speed, which is a good indicator of cognitive function^44^. These results are in line with a previous study that showed that processing speed training improves selective attention in older adults^45^.

### Working memory: Rush Back

Similarly to other CMGs, scores and reaction time of *Rush Back*, which mainly trains working memory, were improved in all age groups with a slower progression in the older groups. It has been demonstrated that older adults can improve their working memory after a specific training^46^. In another study the investigators analyzed the effect of a 20-session training program using an *n*-back task program (same principle as the Rush Back where the subjects must remember the previous card) in younger, middle-aged and older adults^47^. The authors found that age exerted independent effects on training gains and asymptotic performance: older adults tended to show less improvement in scores than younger adults^47^, which is also consistent with our findings.

There are three main limitations in this study: the first is that we did not have access to any information about the background of the participants: it is well-known that several factors influence cognitive function and the risk of dementia such as genetic risk factors^48^, as well as non-genetic risk factors including lifestyle-related factors^49^, for example education level, smoking history, history of hypertension, dyslipidemia, physical activity, body mass index, or concomitant pathologies such as stroke^50^, cardiovascular disease^51^, diabetes^52^, or chronic respiratory disease^53^. Gender is also postulated to influence some cognitive functions such as vocabulary capacity^54^. Due to the fact that we did not have access to this background information, we cannot establish whether the effects observed in the current study were influenced by any of these factors. Most probably, subjects playing with this kind of app are cognitively healthy and quite comfortable with mobile devices.

The second limitation is the choice of the outcomes, namely, the scores of the CMG and the processing speed data obtained within the games. It could be argued that traditional clinical scales or scores would have been more effective in evaluating subjects’ cognitive functioning; however, using the scores of the games to assess the course of the performance has been used in a previous study^55^. Furthermore, both of the scores of the CMG and processing speed have been shown to be good indicators of cognitive function^16,19^. In a recent study examining the effects of cognitive training on cognitive performance of healthy adults, the authors found that there was a transfer effect between the trained abilities and the instruments used only when the tests were similar to the trained situation (near effects). If the tests differed too much from the training tasks (far effects) no training effect was observed^56^. Therefore, using the scores of the games and the reaction times could be considered as a near-effect instrument/measure and quantification of the real transfer to daily activities is still needed. However, some studies did show a transfer to general cognitive function as tested byneuropsychological batteries for multiple cognitive domains^11,16^ and also demonstrated a protective effect in patients with MCI^57^. Those beneficial effects could be related to the multi-domains, novel and continuously challenging (self-adaptative) stimulation provided by most cognitive training apps, which has been shown to be superior to the routine mental activities of everyday life^11^. These challenging and unusual stimuli induce changes in brain activity and connectivity in areas that are known to be affected by aging and neurodegenerative diseases. Those changes may help counteract age- and disease-related alterations and help to explain cognitive benefits and transfers, once their link with cognitive improvements has been clearly established^33,57^.

Finally, the study suffers a selection bias, since the participants were all users of this app and were therefore most probably familiar with the use of smartphones and current technology. This has two consequences: first, older people who are less familiar with mobile technology might find this app less usable and therefore the adherence may be lower. Secondly, a recent study underlined the importance of digital devices use in delaying cognitive decline in the older adults^58^, thus the participants of this study may have already been benefiting from this phenomenon and thus functioning at a higher cognitive level than those who do not regularly use mobile technology. Despite these limitations, the results of this study support that even at old age (above 80 years old), participants are able to use CMG and to train and improve cognition through CMG.

Although technological devices and medical-related apps cannot single-handedly improve cognitive decline, in the absence of effective, low-cost, and accessible treatments for cognitive and motivational deficits, these brain training apps could be greatly beneficial to public health. One salient aspect of the games is that they could be combined with automated evaluation and assessment of cognitive function^16,59^. Therefore, we strongly suggest that the evaluation of cognitive function for long-term follow-up should not be restricted to cross-sectional measurement (typically done only once per year) but should also include longitudinal measurement to evaluate subjects’ learning abilities or cognitive abilities in general if there is no training in between the assessments^15^. In this context, the presented method could be an interesting complementary tool due to its potential to become widely available thanks to the growing use of mobile technology. Another positive aspect is that the cognitive training and follow-up with games on mobile can be also proposed to patients with limited mobility, or living to far to come on a regular basis to specialized centers^60^, and in lockdown during the COVID-19 pandemic^61,62^.

While cognitive training app games have been shown to improve memory in older people with mild cognitive impairment^63^, further studies are needed to determine if technologies, such as apps, can decrease dementia risk in healthy subjects or slow down the progression of the disease in patients suffering from cognitive impairment and if there is a transfer to the activities of daily living. We can, also, speculate that since psychomotor slowing associated with aging has an important negative effect on multi-tasking activities of daily living, improving the processing speed could have a positive effect on the quality of life of the participants^64^.

## Methods

### Study design and participants

We carried out a retrospective observational study in which we obtained anonymized CMG results of healthy participants. This study was approved by The Cambridge Psychology Research Ethics Committee (Pre.2020.28) and research was performed in accordance with relevant guidelines, and informed consent was obtained from the participants and they agreed that their data could be used for research purposes when installing the app. The scores of the CMG, automatically recorded by the application, were then analysed anonymously for each of the five age groups provided: 60–64, 65–69, 70–74, 75–79, and 80 years or older. The number of participants varied in each CMG and in the different age groups (Table 1).

### Procedures

In this study, we used a set of seven individual short CMG provided by Peak brain training (www.peak.net, London—UK) to analyze changes in-game scores and processing speed over the course of 100 sessions of CMG (one session is defined as the completion of one level of the CMG). The games are organized by categories based on the main cognitive functions on which they focus. The seven CMG were selected based on a previous study that identified correlations between CMG scores and scores in two clinically-established cognitive assessments (the Mini-Mental State Examination and Addenbrooke’s Cognitive Evaluation) in older subjects with and without cognitive impairments^16^.

**Figure 3:**
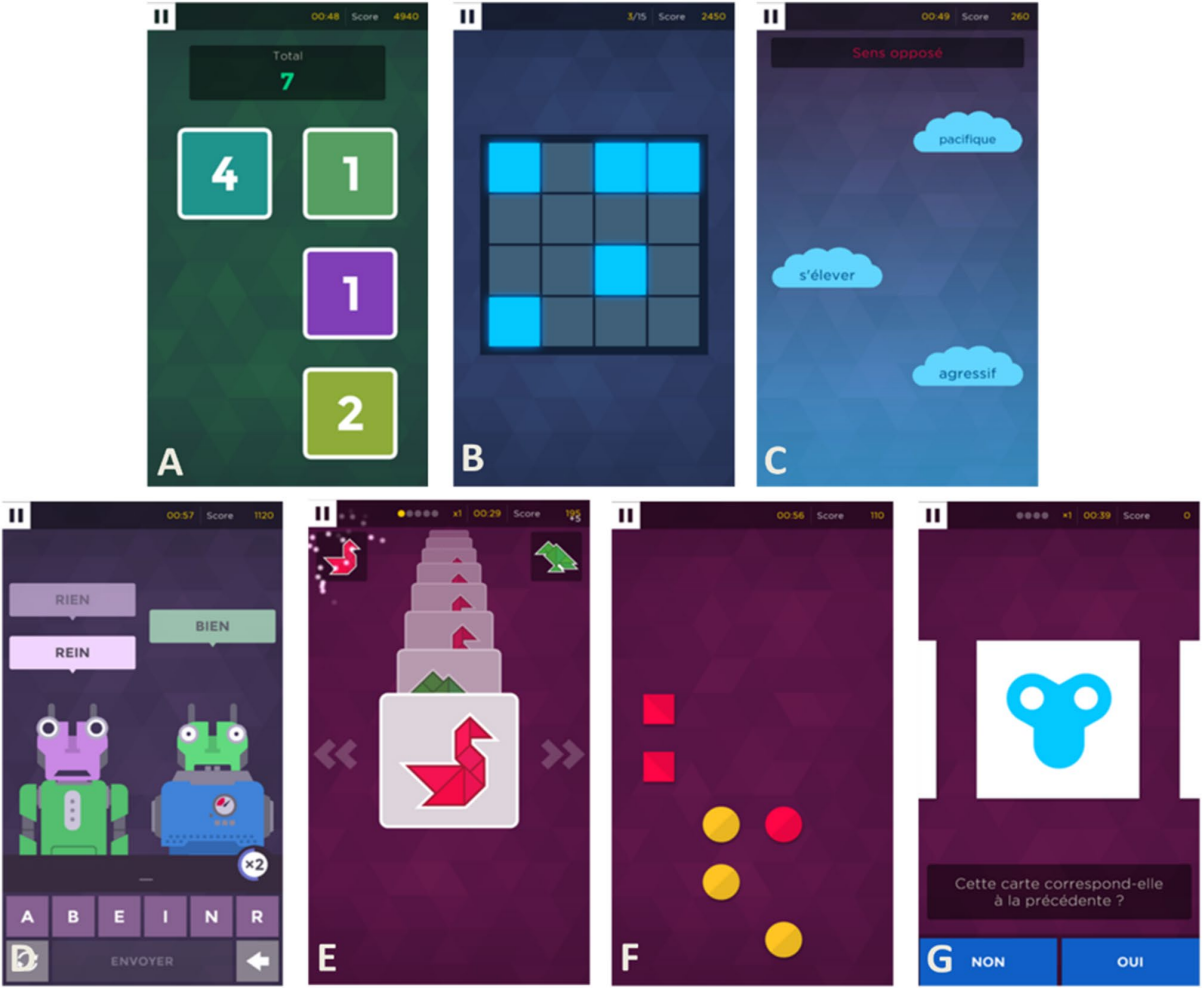
Screenshots of the 7 CMG used in this study. (**A**) Square Numbers, (**B**) Memory Sweep, (**C**) Word Pair, (**D**) Babble Bots, (**E**) Must Sort, (**F**) Unique, (**G**) Rush Back. Instructions and main cognitive abilities trained of each CMG are presented in Table 5.

Screenshots of the games are presented in Fig. 3, and games description and main cognitive abilities trained in Table 5. The difficulty level of each CMG is adapted automatically according to the previous performance of the participant (i.e., rate of correct responses and reaction time). The number of stimuli and the intersimulus intervals depend on the CMG and the difficulty level The CMG were played on smartphones or tablets and the scores of 100 training sessions were analyzed. No particular instructions were given to the participants about the frequency or the duration of each training session, the total duration needed to achieve the 100 sessions of training for the different CMG is presented in Supplementary Table S1.

### Outcomes

The primary outcome was the scores obtained in the seven CMG for the different age groups. Several cognitive sub-functions are usually assessed during standard cognitive evaluations: attention, memory, fluency, language, and visuospatial abilities (Table 5)^65^. To have a complete overview of the cognition, those different sub-functions need to be assessed individually; the scores of the CMG are used as a proxy of the main sub-cognitive abilities challenged in each game.

As a second primary outcome, we computed the processing speed based on the reaction time for the speed-dependent CMG (exceptions were Memory Sweep and Word Pairs)^66^. Details of the computations are presented in Table 4. Processing speed is considered as a good indicator of general cognitive performance^19^ and has been proposed as a predictor of frailty risk among people in old age^67,68^.

### Statistical analysis

Two different kinds of analyses were performed using the CMG score data:

- Firstly, the first session scores of the different age groups were compared using one-way analysis of variance (ANOVA) or Kruskal–Wallis tests, depending the distribution of the data, to determine if age had an influence on the initial scores. Omega-squared analyses or epsilon-squared (non-normally distributed) tests were computed to estimate the effect size^69^. Post-hoc tests for linear trends were performed last.
- We then analysed each CMG using a separate mixed model with random slope (age) and intercept with the scores from each session treated as repeated measures adjusted for the total duration of the training for each participant. Fixed effects of age group, session (1 to 100), and the interaction between age group and session were specified, and the estimated baseline measures were constrained to be identical in the age groups by subtracting the mean values of the first session for each age group in all the sessions.

This approach is equivalent to adjusting for baseline and permitting the relationship between baseline and follow-up scores to differ at each session.

$$ Score_{i,t} = \beta session_{t} + \beta age \,group_{t} + \beta \left( {session \times age\, group} \right)_{t} + \varepsilon_{i,t} + \left( {\alpha + \alpha_{i} } \right), $$

$$ \varepsilon_{i,t} \sim N\left( {0, \sigma^{2} } \right), $$

$$ \alpha_{i} \sim N\left( {0, \mu^{2} } \right), $$

with α and β representing fixed effect, $\varepsilon_{i,t}$ random error and $\alpha_{i}$ the measure of the random effect. Likelihood-ratio tests were used to test the significance of the random effects model and linear mixed model with interaction.

For the processing speed, we applied a separate mixed model for the different CMG with random slope (age) and intercept with the processing speed from each session treated as repeated measures, adjusted for the difficulty levels reached and the total duration of the training for each participant.

Statistical analyses were performed at an overall significance level of 0.05, carried out in RStudio (version 1.1.442), using R version 3.4.4^70^.

## Supplementary Information

Supplementary Information.

## Data Availability

The data that support the findings of this study are available from the corresponding author upon reasonable request.
