# Supplementary material for: Brain training using cognitive apps can improve cognitive performance and processing speed in older adults
Source: Sci Rep. 2021 Jun 10;11:12313. doi: 10.1038/s41598-021-91867-z (PMC8192763; doi:10.1038/s41598-021-91867-z)

BRAIN TRAINING USING COGNITIVE APPS CAN IMPROVE COGNITIVE PERFORMANCE AND PROCESSING SPEED IN OLDER ADULTS

Prof. Bruno Bonnechère, Prof. Malgorzata Klass, Dr. Christelle Langley,

Prof. Barbara Jacquelyn Sahakian

Supplementary materials

**Supplementary Table 1: Median (p25 ; p75) numbers of days needed to achieved the 100 sessions of training for the 7 CMG according to the age of the participants.**

| **Age** | **Square Numbers** | **Memory Sweep** | **Word Pair** | **Babble Bots** | **Must Sort** | **Unique** | **Rush Back** |
| --- | --- | --- | --- | --- | --- | --- | --- |
| *60 – 64* | 517 (237 ; 764) | 606 (405 ; 807) | 426 (225 ; 667) | 174 (73 ; 387) | 410 (236 ; 589) | 402 (222 ; 602) | 477 (306 ; 647) |
| *65 – 69* | 498 (240 ; 759) | 633 (423 ; 824) | 410 (236 ; 642) | 188 (82 ; 394) | 404 (240 ; 596) | 394 (233 ; 602) | 460 (296 ; 642) |
| *70 – 74* | 498 (231 ; 724) | 607 (401 ; 779) | 412 (231 ; 637) | 196 (76 ; 415) | 424 (250 ; 638) | 433 (246 ; 644) | 484 (298 ; 680) |
| *75 – 79* | 482 (202 ; 713) | 576 (351 ; 769) | 390 (211 ; 621) | 233 (93 ; 464) | 387 (214 ; 599) | 399 (213 ; 598) | 448 (269 ; 637) |
| *≥ 80* | 502 (217 ; 785) | 687 (479 ; 888) | 392 (209 ; 653) | 228 (80 ; 496) | 434 (236 ; 627) | 454 (261 ; 660) | 481 (299 ; 676) |
| *Kruskal-Wallis* | χ²(4) = 5.2, p = .26 | χ²(4) = 12.5, p = .013 | χ²(4) = 5.9, p = .21 | χ²(4) = 22.4, p < .001 | χ²(4) = 7.8, p = .11 | χ²(4) = 20.7, p < .001 | χ²(4) = 6.8, p = .14 |

**Supplementary Table 2: β coefficient [95% CI] for the regression between the duration of the training and the normalized progress**

|  | **Square Numbers** | **Memory Sweep** | **Word Pair** | **Babble Bots** | **Must Sort** | **Unique** | **Rush Back** |
| --- | --- | --- | --- | --- | --- | --- | --- |
| *Intercept* | 129  [120 ; 138] | 31  [26 ; 36.6] | 810  [756 ; 864] | 411  [376 ; 446] | 2586  [1868 ; 3303] | 895  [806 ; 984] | 368  [332 ; 405] |
| *60 – 64* | -0.01  [-0.03 ; -0.00] | 0.004  [-0.005 ; 0.014] | -0.09  [-0.22 ; 0.03] | -0.11  [-0.27 ; 0.05] | 1.00  [-0.85 ; 2.86] | -0.17  [-0.40 ; 0.05] | -0.12  [-0.20 ; -0.03] |
| *65 – 69* | -0.03  [-0.05 ; -0.01] | 0.003  [-0.005 ; 0.013] | -0.22  [-0.36 ; -0.09] | -0.07  [-0.23 ; 0.09] | 1.73  [-0.09 ; 3.55] | -0.21  [-0.43 ; 0.01] | -0.12  [-0.21 ; -0.04] |
| *70 – 74* | -0.04  [-0.06 ; -0.02] | -0.003  [-0.013 ; 0.006] | -0.20  [-0.33 ; -0.07] | -0.10  [-0.25 ; 0.05] | 0.91  [-0.84 ; 2.66] | -0.30  [-0.51 ; -0.07] | -0.16  [-0.23 : -0.08] |
| *75 – 79* | -0.05  [-0.08 ; -0.02] | -0.012  [-0.025 ; 0.001] | -0.13  [-0.32 ; 0.05] | -0.08  [-0.23 ; 0.07] | 0.57  [-1.90 ; 3.04] | -0.55  [-0.86 ; -0.24] | -0.19  [-0.30 ; - 0.08] |
| *≥ 80* | -0.03  [-0.06 ; 0.01] | -0.013  [-0.028 ; 0.002] | -0.29  [-0.52 ; -0.05] | -0.18  [-0.32 ; -0.03] | 0.63  [-2.34 ; 3.60] | -0.53  [-0.88 ; -0.17] | -0.14  [-0.27 ; -0.01] |
| *p-value* | F(13,321) = 5.2, p < .001 | F(8,963) = 4.07, p < .001 | F(12,701) = 11.59, p < .001 | F(5,036) = 1.5, p = .18 | F(12,909) = 0.76, p = .79 | F(12,420) = 4.70, p = .001 | F(12,716) = 4.07, p < .001 |

**Supplementary Table 3: Results of the mixed models, β (SE) representing the change of score of the CMG per training session**

| **Parameters** | | **Cognitive Mobile Game** | | | | | | |
| --- | --- | --- | --- | --- | --- | --- | --- | --- |
|  |  | **Square Numbers** | **Memory Sweep** | **Word Pair** | **Babble Bots** | **Must Sort** | **Unique** | **Rush Back** |
| **Fixed effect** | **Session** | 107 (0.6) | 76 (0.3) | 143 (0.5) | 60 (0.9) | 172 (0.5) | 148 (0.4) | 101 (0.3) |
|  | **Age-461 (186)** | | | | | | | |
|  | *65 – 69* | -760 (342) | -1,022 (152) | -419 (241) | -593 (361) | -461 (189) | -1,130 (209) | -935 (121) |
|  | *70 – 74* | -3,561 (351) | -2,255 (170) | -874 (241) | -1,121 (362) | -888 (187) | -2,376 (218) | -1,919 (122) |
|  | *75 – 79* | -5,470 (533) | -3,204 (241) | -1,797 (326) | -2,648 (362) | -1,418 (247) | -4,017 (275) | -2,811 (164) |
|  | *≥ 80* | -6,740 (710) | -3,973 (267) | -2,177 (414) | -2,233 (363 | -1,450 (300) | -3,653 (343) | -2,879 (206) |
|  | **Age * Session** | | | | | | | |
|  | *65 – 69* | -18 (0.9) | -12 (0.4) | -13 (0.6) | 0.16 (1.32) | -42 (0.7) | -25 (0.6) | -14 (0.3) |
|  | *70 – 74* | -31 (1.1) | -21 (0.4) | -28 (0.7) | -7.93 (0.8) | -76 (0.7) | -48 (0.6) | -22 (0.4) |
|  | *75 – 79* | -42.3 (1.5) | -30 (0.7) | -49 (0.9) | -11.55 (1.3) | -102 (0.9) | -67 (0.7) | -27 (0.5) |
|  | *≥ 80* | -44.1 (1.9) | -37 (0.9) | -54 (1.1) | -9.36 (1.1) | -84 (1.1) | -66 (0.9) | -32 (0.6) |
|  | **Intercept** | 29,181 (222) | 34,772 (104) | 10,157 (171) | 11,317 (256) | 6,427 (132) | 13,868 (148) | 18,144 (85) |
| **Random effect** | **μ** | 234,267,648 (15,306) | 33,628,025 (5,799) | 100,217,019 (10,011) | 62,980,880 (7,936) | 59,058,966 (7,685) | 75,492,836 (8,689) | 25,134,036 (5,013) |
|  | **σ** | 108,398,766 (10,411) | 13,448,643 (3,667) | 53,318,302 (7,302) | 67,401,345 (8,210) | 53,277,559 (7,299) | 36,329,895 (6,027) | 14,611,978 (3,923) |

Supplementary Figure 1: Regression between the duration of the training and the progress for Square Numbers.


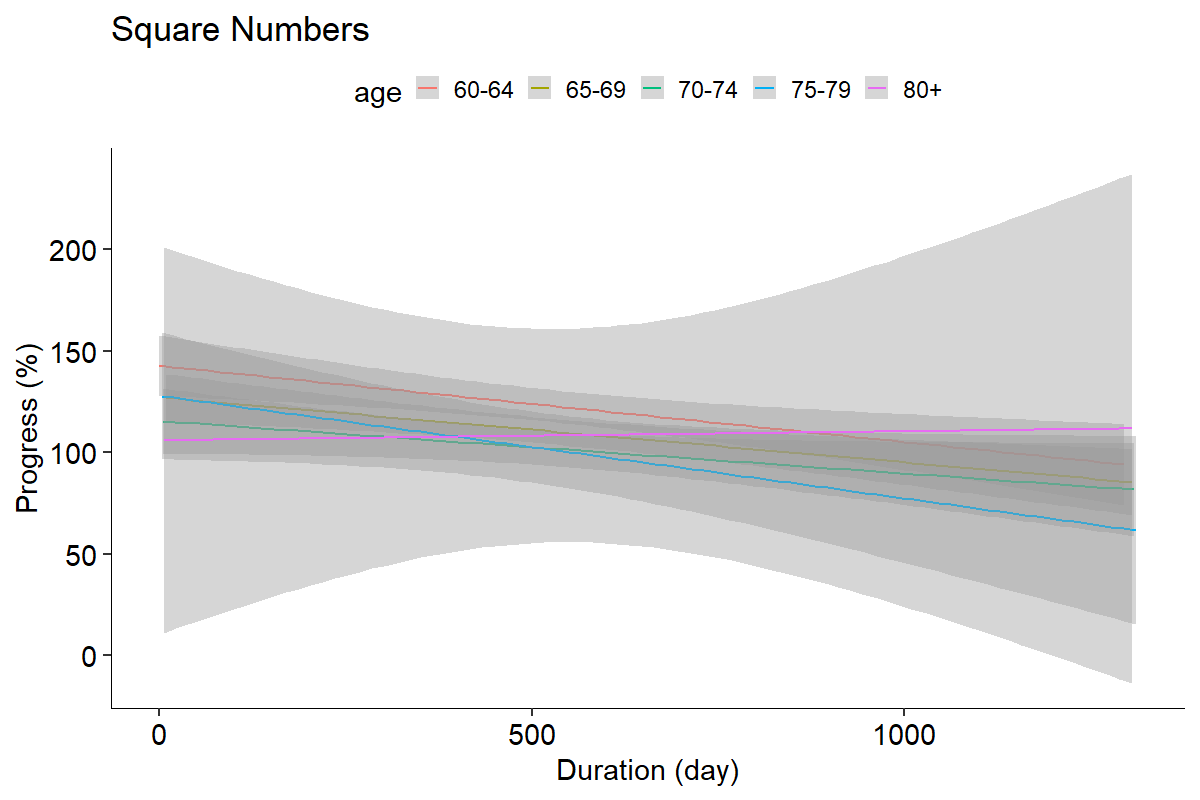


Supplementary Figure 2: Regression between the duration of the training and the progress for Memory Sweep.


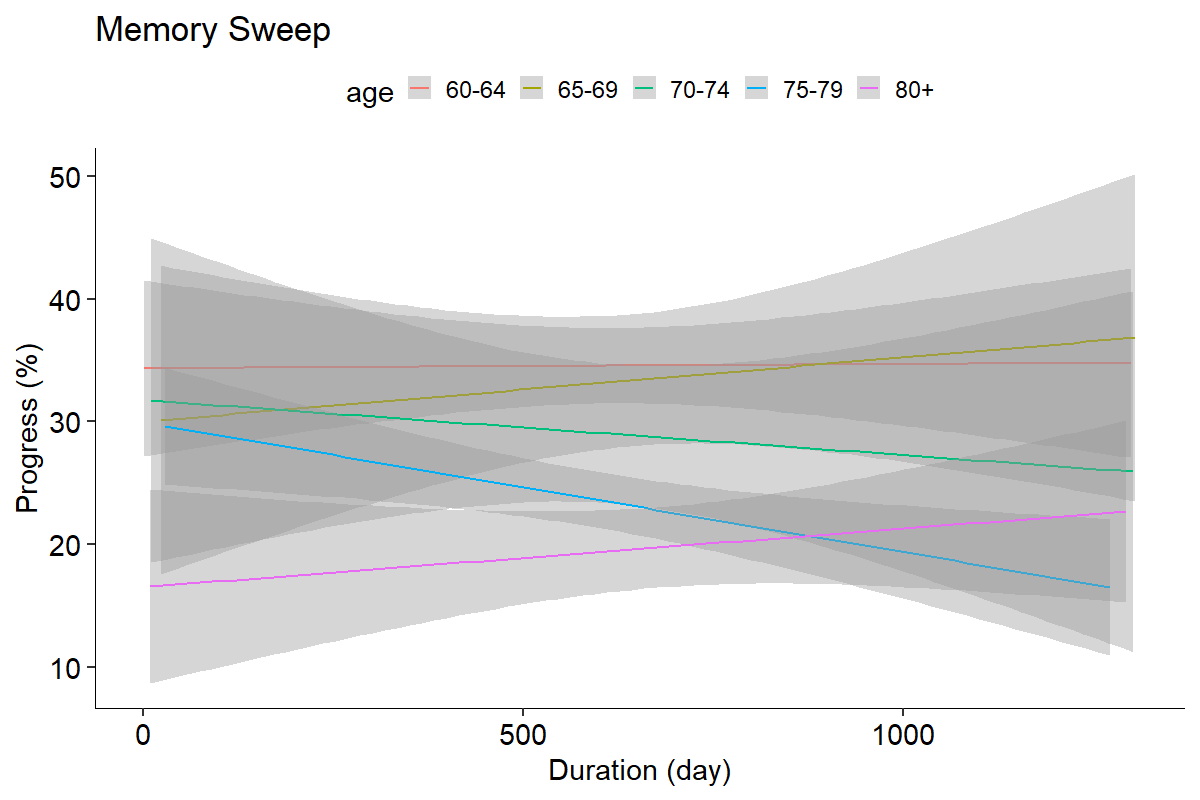


Supplementary Figure 3: Regression between the duration of the training and the progress for Word Pair.


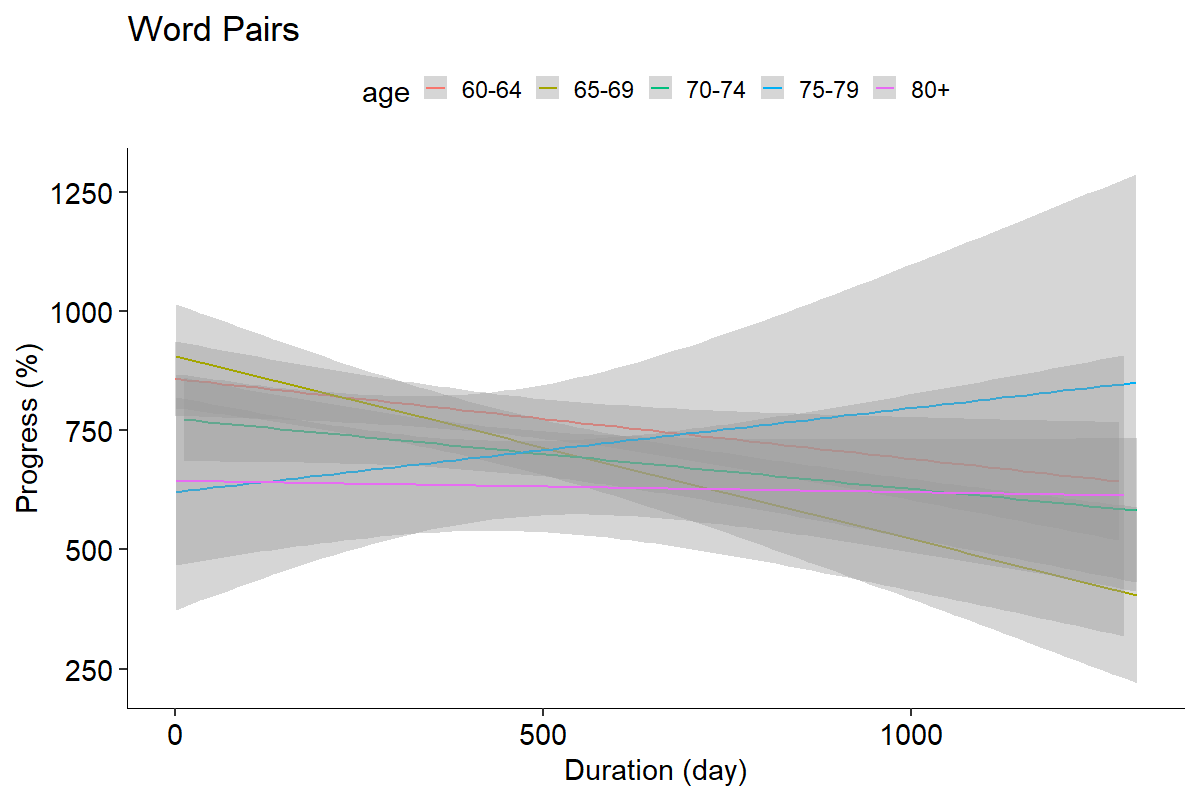


Supplementary Figure 4: Regression between the duration of the training and the progress for Babble Bots.


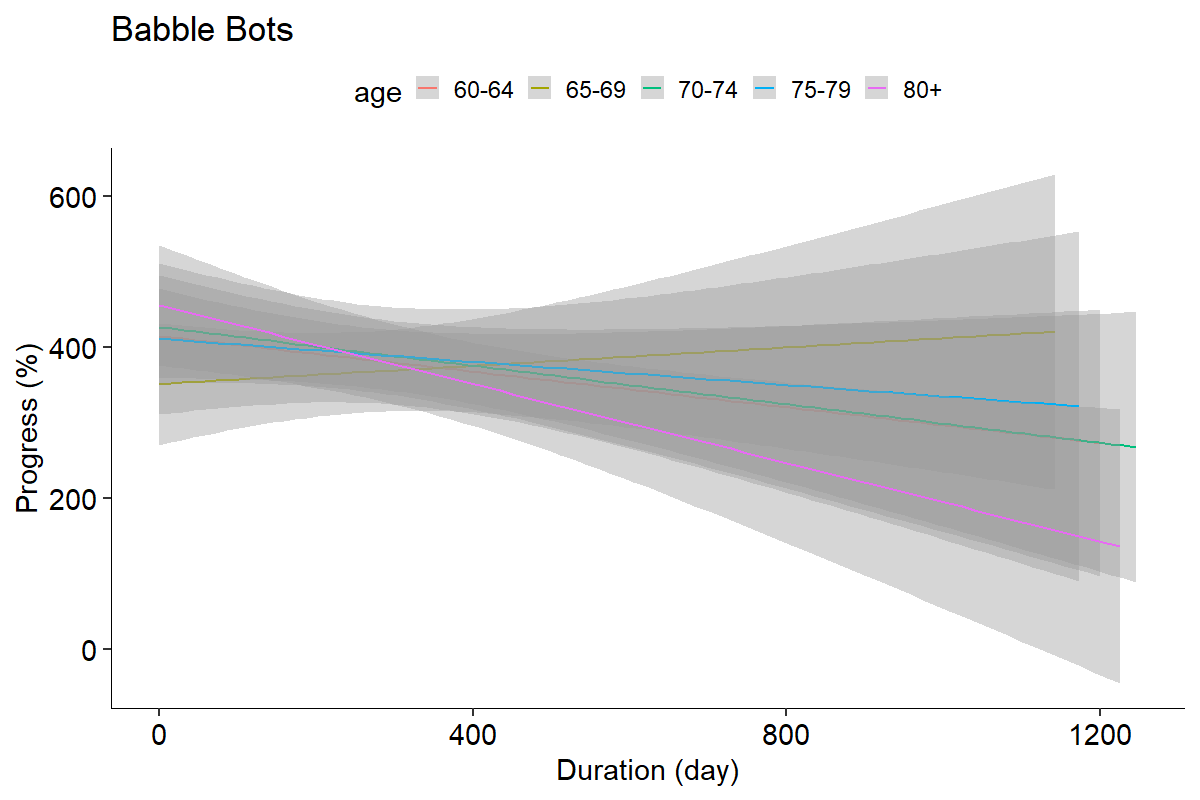


Supplementary Figure 5: Regression between the duration of the training and the progress for Must Sort.


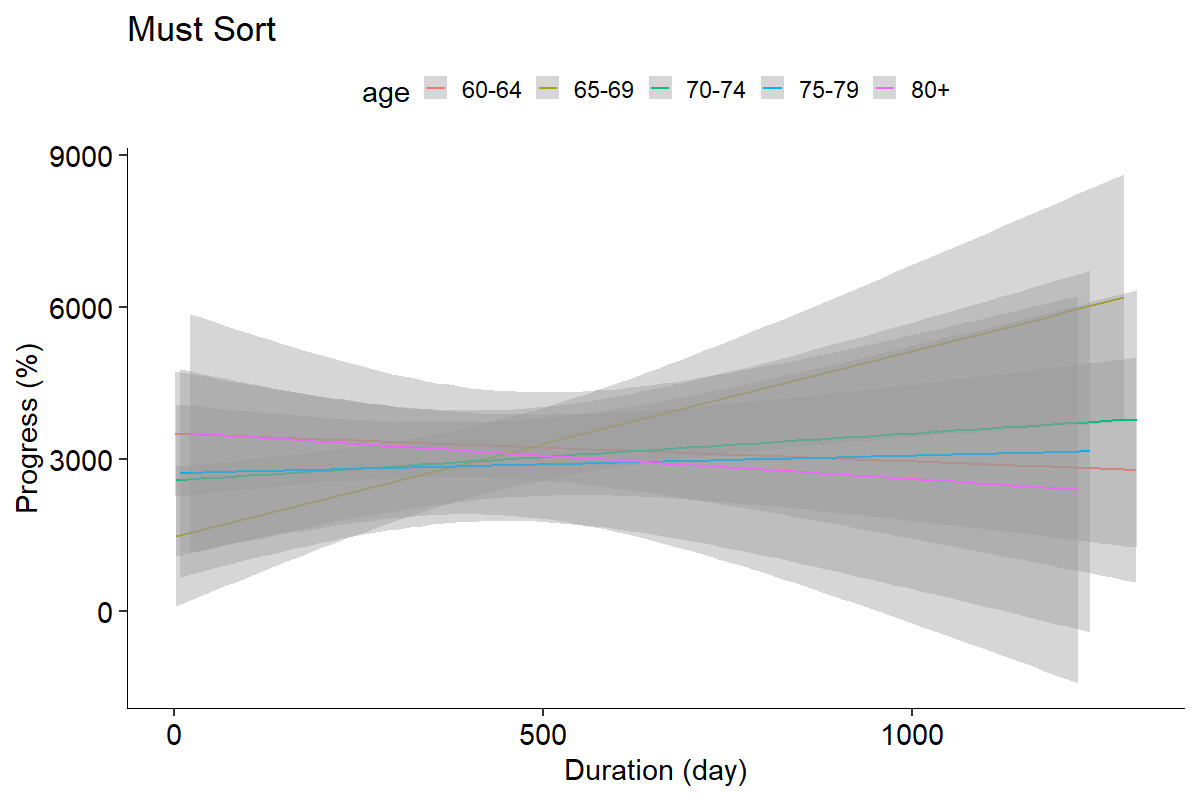


Supplementary Figure 6: Regression between the duration of the training and the progress for Unique.


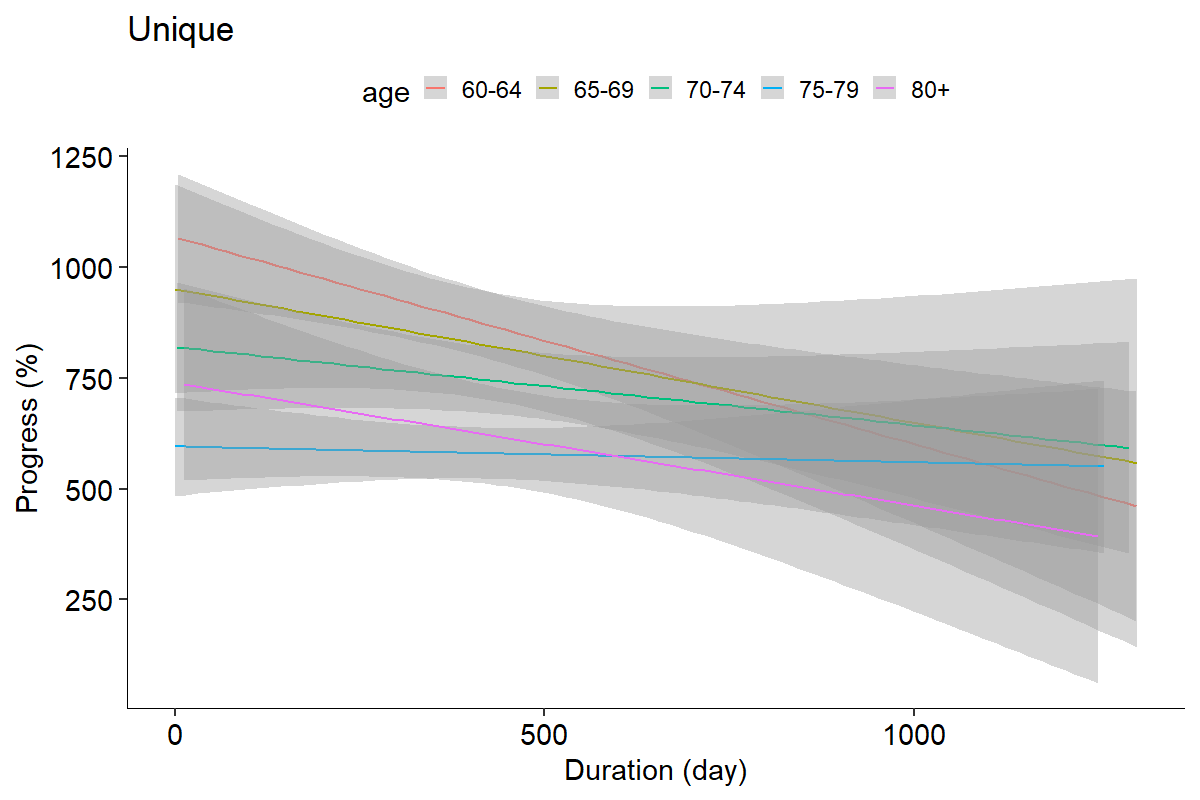


Supplementary Figure 7: Regression between the duration of the training and the progress for Rush Back.


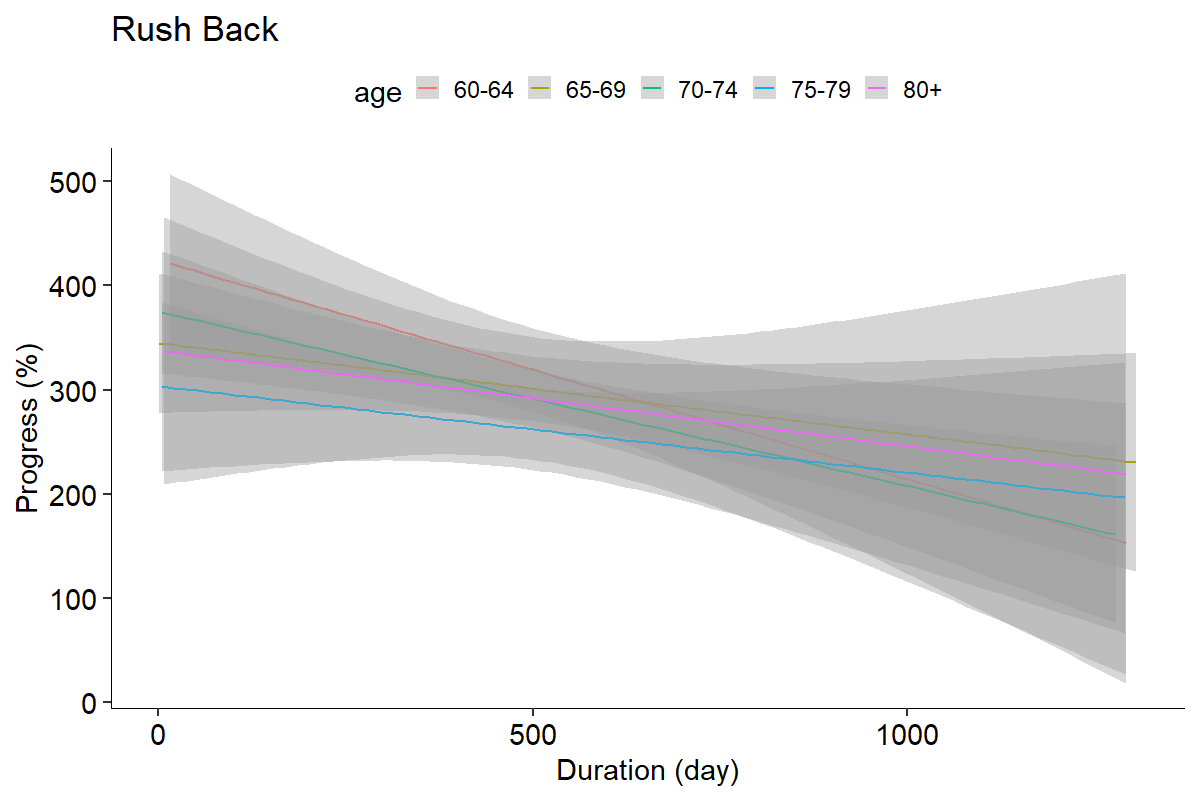

Supplement: Supplementary file 1 — Supplementary Information. [file 41598_2021_91867_MOESM1_ESM.docx]
